# Revealing the Unknown: Real-Time Recognition of Galápagos Snake Species Using Deep Learning

**DOI:** 10.3390/ani10050806

**Published:** 2020-05-06

**Authors:** Anika Patel, Lisa Cheung, Nandini Khatod, Irina Matijosaitiene, Alejandro Arteaga, Joseph W. Gilkey

**Affiliations:** 1 Data Science Institute, Saint Peter’s University, Jersey City, NJ 07306, USA; anikapatel2302@gmail.com (A.P.); lcheung@saintpeters.edu (L.C.); nandinikhatod@yahoo.in (N.K.); jgilkey@saintpeters.edu (J.W.G.J.); 2 Institute of Environmental Engineering, Kaunas University of Technology, 44249 Kaunas, Lithuania; 3 Department of Information Technologies, Vilnius Gediminas Technical University, 10223 Vilnius, Lithuania; 4 Tropical Herping, Quito 170150, Ecuador; af.arteaga.navarro@gmail.com

**Keywords:** artificial intelligence (AI) platform, deep learning, Galápagos Islands, image classification, machine learning, *Pseudalsophis*, racer snake, region-based convolutional neural network (R-CNN), snake species

## Abstract

**Simple Summary:**

The snakes in Galápagos are the least studied group of vertebrates in the archipelago. The conservation status of only four out of nine recognized species has been formally evaluated, and preliminary evidence suggests that some of the species may be entirely extinct on some islands. Moreover, nearly all park ranger reports and citizen/science photographic identifications of Galápagos snakes are spurious, given that the systematics of the snakes in the archipelago have just recently been clarified. Our solution is to provide park rangers and tourists with easily accessible applications for species identification in real time through automatic object recognition. We used deep learning algorithms on collected images of the snake species to develop the artificial intelligence platform, an application software, that is able to recognize a species of a snake using a user’s uploaded image. The application software works in the following way: once a user uploads an image of a snake into the application, the algorithm processes it, classifies it into one of the nine snake species, gives the class of the predicted species, as well as educates users by providing them with information about the distribution, natural history, conservation, and etymology of the snake.

**Abstract:**

Real-time identification of wildlife is an upcoming and promising tool for the preservation of wildlife. In this research project, we aimed to use object detection and image classification for the racer snakes of the Galápagos Islands, Ecuador. The final target of this project was to build an artificial intelligence (AI) platform, in terms of a web or mobile application, which would serve as a real-time decision making and supporting mechanism for the visitors and park rangers of the Galápagos Islands, to correctly identify a snake species from the user’s uploaded image. Using the deep learning and machine learning algorithms and libraries, we modified and successfully implemented four region-based convolutional neural network (R-CNN) architectures (models for image classification): Inception V2, ResNet, MobileNet, and VGG16. Inception V2, ResNet and VGG16 reached an overall accuracy of 75%.

## 1. Introduction

Despite being noteworthy inhabitants of the world’s most widely studied living laboratory, the snakes in Galápagos are the least studied group of vertebrates in the archipelago. The conservation status of only four out of the nine recognized species has been formally evaluated [1,2,3,4], and preliminary evidence [5] suggests that some of the species may be extinct in some islands despite being evaluated as of “Least Concern” by IUCN (International Union for Conservation of Nature) assessments. Perhaps the most important reason why some of these local extinctions have gone unnoticed in Galápagos, one of the world’s capitals of wildlife-oriented tourism, is because nearly all park ranger reports and citizen-science photographic identifications of Galápagos snakes are erroneous. This is not surprising, given that the systematics of the snakes in the archipelago has just recently been clarified [6], and even now, it remains obscure and out-of-reach for the general public. One way to cope with this challenge is to provide park rangers and tourists with easily accessible applications for species identification through automatic object recognition.

Real-time image identification of snake species is an upcoming and promising solution to bridging the reptile taxonomic gap, which receives considerable attention in both data science and herpetologist communities. Wildlife experts explore different regions of the world to find these legless creatures and observe them in their natural habitat. Taxonomists have been searching for more efficient methods to meet species identification requirements, such as developing digital image processing and pattern recognition techniques [7] and using objection detection by camera traps that will precisely and concisely identify species [8]. Object recognition in images is a relatively new area; the first (deep) convolutional neural network architecture and early achievements in text document recognition were described by LeCun in 1998 [8]. More recent deep neural networks perform well in face recognition and object detection in streets, airports, and other buildings due in large part to the high volume of images that are available to train the models (hundreds of thousands of images). Therefore, having a small number of available images forces researchers to apply creative approaches by modifying the architecture of existing neural networks or developing their own neural network architecture. The novelty of our research is highlighted by the creative modification and application of novel state-of-the-art deep convolutional neural network architectures that have been trained by our team on a small number of images and received a high model performance. Moreover, due to a considerably short image testing time, our proposed artificial intelligence (AI) platform for the real-time recognition of the Galápagos snake species would be the first of this type and may be a successful mobile/web application for snake species recognition in one of the most protected and diverse national parks on Earth.

Szabolcs Sergyán, in 2007 [9], implemented a color content-based image classification where color is stored in the intensity vectors of image pixels and the information can be retrieved easily. Color can be represented in different color spaces or features and can be stored in several ways. RGB space is a widely used color space for image display. It is composed of three color components: red, green, and blue (Table 1).

YCbCr is a family of color spaces used in video systems. Y is the luma component and Cb and Cr are the blue and red chroma components, respectively. HSV (hue, saturation and value) space is frequently used in computer graphics. The three-color components are hue, saturation (intensity), and value (brightness). Similarly, for the color-based features, the most commonly used descriptors are color moments, color histograms, and color coherence vector (CCV). Szabolcs Sergyán implemented k-means clustering, where he classified images into four clusters (k = 4) using the obtained six features (4 from moments, 1 from histogram, and another 1 from color coherence vectors) per color space.

**Figure 1:**
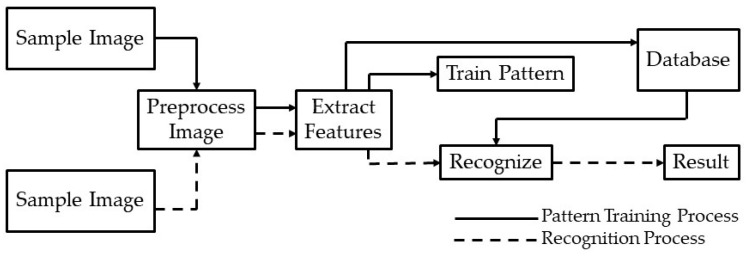
System architecture for feature extraction and pattern recognition used by Hernandex-Serna and Jimenez-Segura to identify different species of fish, butterflies, and plants [10].

In 2014, Andres Hernandex-Serna and Luz Fernanda Jimenez-Segura [10] used an automatic object detection with the use of photographic images to identify different species of fish, butterflies, and plants. This included 697 fish species, 32 plant species, and 11 species of butterflies that were taken from the Natural History Museum records and online. They implemented feature extraction and pattern recognition to build their detection models. The images were preprocessed using the GrabCut algorithm to remove noisy backgrounds, so feature was extracted easily. Fifteen features were extracted and fed into an artificial neural network. A system architecture (Figure 1) [10] was created to define the process of when an image is fed into the model and how that model will recognize it. A sample image is given to the model, the model preprocesses the image and then extracts its features. If the model recognizes the image, it will provide a result. However, if it is unrecognized, the image will be trained and stored into its memory before giving the result. Their work shows promising results, with up to 90–91.65% of true positive fish identifications, 92.87% of plants, and 93.25% of butterflies. However, a challenge they face is phenotypes that are similar and harder to distinguish.

Relevant research has also been performed by the University of Malaysia Perlis on snake species identification using machine learning techniques [11]. The team of researchers has gathered 349 samples of 22 species of snakes from Perlis snake park in Malaysia and created a database called Snakes of Perlis Corpus. They used textural features extraction as their key method in identifying the snake species. To extract the textural features, CEDD (color and edge directivity descriptor) was used, which incorporates color and texture information in histograms. Features are created using two steps. First, the histogram is divided into six regions based on texture. Second, 24 regions are calculated from each of these regions by using color characteristics. After extracting the textural information using CEDD, they implemented five machine learning algorithms to classify the snake images: naïve Bayes, nearest neighbors, K-nearest neighbors, decision tree J48, and backpropagation neural network. The nearest neighbors algorithm uses unsupervised learning and starts with k = 1. It is unsupervised because the points have no external classification. On the other hand, K-nearest neighbors (K-NN) is a supervised learning classification. It classifies a point based on the known classification of other points. Backpropagation neural network and K-nearest neighbors gave the best accuracy and precision (99% and 96%, respectively) (Table 2).

Kody G. Dantongdee and Franz Kurfess from California Polytechnic State University [12], San Luis Obispo, built an image classifier to identify plants. The dataset for this research was produced by searching Google Images using Python script adapted from a web-crawler created by Hardik Vasa [13]. Convolutional neural networks (CNN), commonly used for image classification, were applied here to classify the plants. The model was trained using the dataset at the genus-species level, which had approximately 100 images per class. The model used for classification was a very basic inception model of CNN. The design of these networks is made up of a series of convolutional, pooling, and fully connected layers. Although they failed to achieve good accuracy on the plant classifier due to an incomplete dataset and basic CNN design, the research gave good scope for future researchers to customize designs of neural networks that can be implemented and used by scientists or potential users in the botanical field through mobile applications.

**Figure 2:**
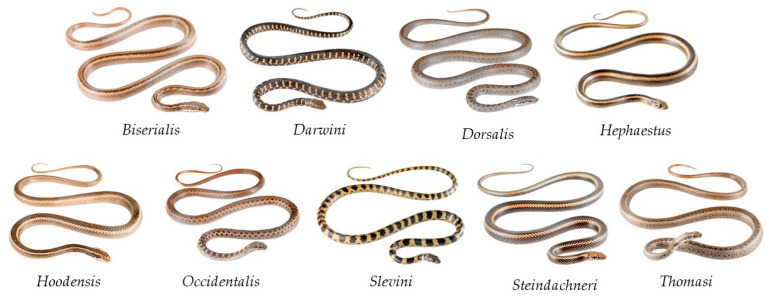
*Pseudalsophis* snake species found in the Galápagos Islands, Ecuador, and used in this research [14].

Snake species identification may be useful for wildlife monitoring purposes. Images captured by camera traps in national parks and forests can be used to assess the movement patterns of the corresponding reptiles. In this paper we present the different deep learning convolutional neural networks used to identify a set of snake species found in the Galápagos Islands. The Ecuadorian institution Tropical Herping provided the image dataset of 9 *Pseudalsophis* snake species (Figure 2).

## 2. Materials and Methods

The final goal of this research was to develop an artificial intelligence platform for the real-time object recognition of snake species in the Galápagos islands by applying machine learning and deep learning techniques on snake images. This requires two main tasks: object detection and classification of images of the *Pseudalsophis* snake species. R-CNN (region-based convolutional neural network) is widely used for object detection, whereas CNN (convolutional neural network) is used for image classification. 

### 2.1. R-CNN Structure

R-CNN works with the feature extraction process. It has three main layers of operations: feature network, region proposal network (RPN), and detection network [15,16,17]. The function of the feature network is to extract the features from the image and output the original shape and structure of the mapped image to the pixel coordinates of the image. The region proposal network has three convolutional layers from which one is used for classification and the other two are used for finding the regions that contain the feature pixels that make up the image. The purpose of the RPN is to generate the bounding boxes called regions of interest (ROI) that have higher probability of containing an object [15,16]. The detection network takes the input from the feature network and RPN and generates the final class and bounding box, which are fully connected layers. Training is performed at the RPN and detection layers.

#### 2.1.1. Training the RPN

**Figure 3:**
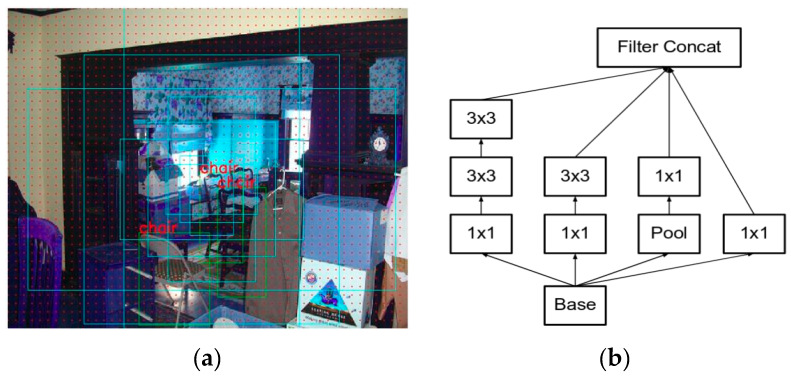
(**a**) Anchor boxes generated around each of nine anchors [15]. (**b**) Inception V2 module in which the 5 × 5 convolutions are replaced by two successive 3 × 3 convolutions and pooling is applied [20].

For training the RPN, bounding boxes are generated by mechanisms known as anchor boxes (Figure 3). Every pixel of an image is considered as an anchor. Nine rectangular boxes of different shapes and sizes are generated around each anchor (due to 9 proposals generated for each pixel in the image: 3 aspect/ratio and 3 scale), hence there are tens of thousands of boxes around each anchor in the image [15,17,18,19]. To remove the multiple boxes around each anchor, the algorithm applies a reduction operation called a non-maximum suppression. It removes the overlapping boxes on each object by considering a box with the highest score. Thus, during the training phase of RPN the number of boxes is reduced to 2000 [15]. During the testing phase, the score is fed to the detection network to indicate which boxes to keep.

Then, the RPN predicts if the anchor is background or foreground and refines the anchor. After determining the bounding boxes from all the above processes, there is a need to label the anchor, for which the ground truth boxes are used. The anchor that largely overlaps with the ground truth as foreground and the one that overlaps less as background are then labeled [15,16], hence, the classification task is implemented at this step.

In the above described method, loss occurs with the background anchor that is not compared with the ground truth boxes. Therefore, to define these background anchors and to minimize the error, a smooth-L1 loss on the position (x, y) of the top-left box is used, as well as the logarithm of the heights and widths [16]. The overall loss of the RPN is a combination of the classification loss and the regression loss, since the RPN has a classifier to determine the probability of a proposal having the target object, and a regressor to regress the coordinates of the proposals.

$$L_{loc}\left(t^{u},v\right)=\underset{i\in \left\{x,y,w,h\right\}}{\sum }smooth_{L1\;}(t_{i}^{u}-v_{i),}$$

where $smooth_{L_{1}}\left(x\right)=\{\begin{array}{c} 0.5\;x^{2}\;if\;\left|x\right|<1\\ \left|x\right|-0.5\;otherwise \end{array}$.

#### 2.1.2. Training the Detection Layer

Training the detection layer is similar to training the RPN. First the 2000 of intersections over unions (IOUs) generated by RPN against ground truth are calculated. Then, ROIs are labeled as foreground and background depending on the threshold values. Then, a fixed number of ROIs are selected from the foreground and background. Features are cropped and scaled to 14 × 14 [17,18,19]. The set of cropped features for each image is passed through the detection network as a batch. The final dense layers are created for each cropped feature, with the score and bounding box for each class.

To generate the label for the detection network classification task, IOUs of all the ROIs and the ground truth boxes are calculated depending on the IOU thresholds (e.g., foreground above 0.5, and background between 0.5 and 0.1), thus, labels are generated for a subset of ROIs. The difference with RPN is that it contains more classes.

### 2.2. R-CNN Implementation

Implementation of different modified architectures of R-CNN let us test and determine the best fit method to correctly identify species of snakes: Inception V2, ResNet, MobileNet, and VGG16.

#### 2.2.1. Inception V2

While processing the image in the neural network, the inception module prevents the model from overfitting. Images of different sizes are filtered at the same level thus making the network wider rather than making it deeper. The model “uses convolutions of different sizes to capture details at varied scales (5 × 5, 3 × 3, 1 × 1)” [20,21]. We applied the Inception V2 module in which 5 × 5 convolutions are replaced by two successive 3 × 3 convolutions and applied pooling as shown in Figure 3b. The inception model also replaced the fully connected layers at the end with the pooling layer, where max pooling is performed and the 2D feature map that reduces the number of parameters is obtained. Inception V2 has 22 layers, where going deeper with convolutions led to a smarter approach: Instead of stacking convolutional layers, the authors of Inception V2 stack modules, within which the convolutional layers are located [22,23].

#### 2.2.2. ResNet

**Figure 4:**
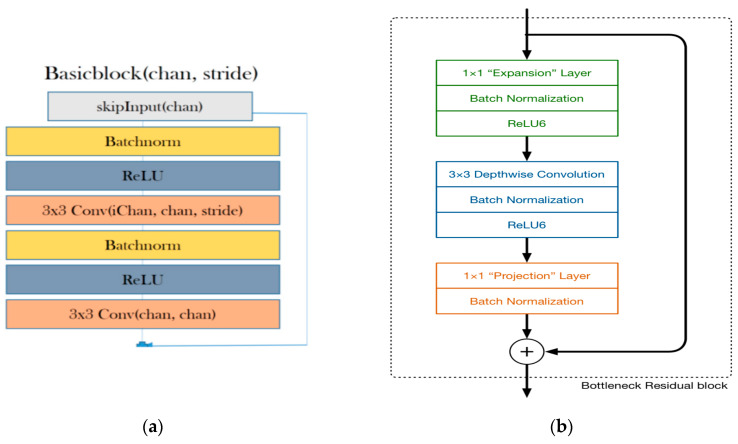
(**a**) ResNet architecture [21]. ReLU is a linear rectifier function, which is an activation function for the neural network. Batchnorm, or batch normalization, is a technique used to standardize the input either by an activation function or by a prior node. Stride is the number of pixel shifts over the input matrix. For instance, when the stride is 1, then we move the filters 1 pixel at a time, and when the stride is 2, then we move the filters 2 pixels at a time. (**b**) MobileNetV2 architecture [26].

ResNet was designed by the Microsoft Research team, where they address the problem of decreasing accuracy with the increase of depth of neural networks by using skip connections (such as residuals), while actually making the models deeper [24]. The key insight into the stability of ResNet is that the architecture tries to model the residual of the intermediate output, instead of the traditional method of modeling the intermediate output itself [25]. This allows researchers to train ResNet models that are comprised of hundreds of layers. We propose a modification in the ResNet architecture as described in Figure 4. Our proposed ResNet model comprises basic blocks that are, in turn, derived from a pair of convolutional layers that are pre-processed using batch normalization and are passed through a non-linear rectifier function. The aim is to compute an exponentially increasing number of independent representations at each basic block. In contrast, the traditional ResNet only increases the number of intermediate representations from 16 up to 64 across the entire network.

#### 2.2.3. MobileNet

The MobileNetV2 architecture is based on an inverted residual structure where the input and output of the residual block are within bottleneck layers, in contrast to traditional residual models that use expanded representations in the input. MobileNetV2 uses lightweight depth-wise separable convolutions to filter features in the intermediate expansion layer (Figure 4b) [27].

#### 2.2.4. VGG16

**Figure 5:**
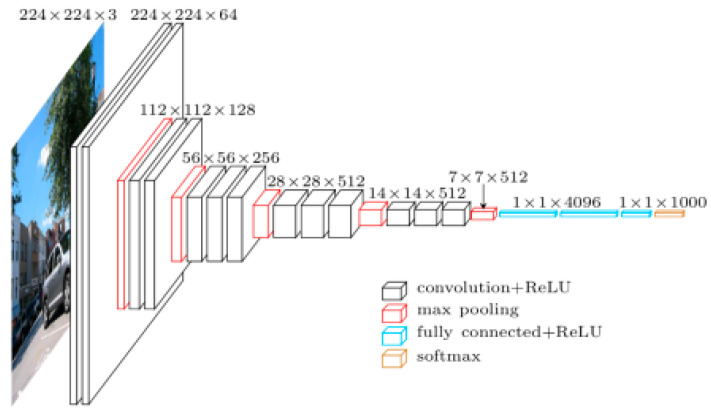
VGG16 architecture [26]. ReLU is a linear rectifier function, which is an activation function for the neural network. Softmax is the final layer of the neural network that has a value either 0 or 1.

VGGNet is another convolutional neural network model that was created by Karen Simonyan and Andrew Zisserman at Oxford Visual Geometry Group. The network is simple compared to most convolutional neural networks and works with a simple 3 × 3 stacked layer [28]. The application of this model is available due to the large image dataset (ImageNet) it was pretrained on, which has over 14 million images and 1000 different classes of images. There are two different VGG (Visual Geometry Group) models that are available: VGG16 and VGG19. The difference between the two is the number of weight layers. VGG-16 has 13 convolutional and 3 fully-connected layers, with the ReLu function applied to all 16 layers (Figure 5). For our research, VGG16 was implemented.

The model requires a 224 × 244 RGB image to be inputted as the training data. The architecture of the network has three different parameters for a convolutional layer: receptive field size, stride, and padding. The stride refers to how pixels are controlled around convolutional filters for a given image. VGGNet uses a stride of 1 which means the network filters one pixel at a time [29]. Padding describes adding extra pixels outside of the image to prevent images from decreasing in size each time a filter is used on an image [29]. A padding of 1 was used to maintain the same size spirally, since our receptive field was 3. Along with the 3 × 3 convolutional layer, it also has a pooling layer of 2 × 2 with a stride of 2 and zero padding [28].

The VGGNet training procedure is similar to the procedure presented by Alex Krizhevsky’s team for a deep convolutional neural network in the sense that it is “optimizing the multinomial logistic regression objective using mini-batch gradient descent (based on back-propagation with momentum)” [28].

### 2.3. Workflow towards Training the Image Classifier

**Figure 6:**
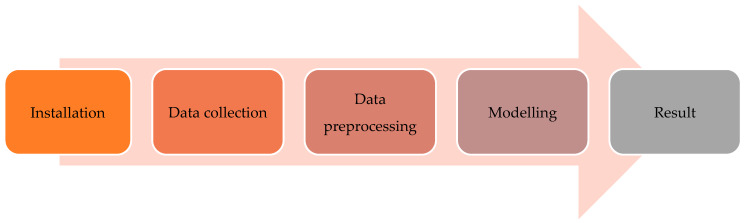
Process of training the image classifier.

Before implementing the R-CNNs we developed the workflow for training the image classifier to correctly identify *Pseudalsophis* snake species (Figure 6). Each step is discussed in the subsections below.

#### 2.3.1. Installation of Libraries

For a machine to learn, a larger sample is preferred. The more images the machine learns from, the easier and quicker it will learn to recognize the species. Therefore, for this research, the machine needs a larger processing and computational power. TensorFlow-GPU allows the computer to use a video card to provide extra processing power while training the model. Thus, a TensorFlow-GPU environment was set up in our local personal computer (PC) that had a NVIDIA GEFORCE GTX 1050 graphics card supporting TensorFlow-GPU version 1.12 along with CUDA version 10 required for image processing in the Windows 10 operating system.

#### 2.3.2. Data Collection

After setting up the environment, we collected our dataset from three different sources. First, from the Tropic Herping collection of images [5]. Second, the images were collected by searching Google images using a Python script adapted from a web-crawler created by Hardik Vasa [13], in a similar process as the image collection completed for the project conducted by Kody G. Dantongdee from California Polytechnic State University, San Luis Obispo [12]. However, some of the images returned in the searches were not correctly identified. Third, we leveraged Flickr [30] for images using the snakes’ genre and species names.

#### 2.3.3. Data Preprocessing

For this project, we had in total 247 images of snakes making up 9 species. These images were manually checked for accuracy in terms of representing the correct snake species. Because raw images cannot be fed to the neural network for training the model, they needed preprocessing, therefore, we performed the following steps: (1) Applied the labeling software to label each of the images. After an image was labeled the xml file for each image was generated and saved in the folder. (2) Converted all xml files to csv files using the Python code. Thus, for training the model, we used the csv files as external parameters (input) to the network (Table 3). As internal parameters for training the models we used a default parameter for the number of layers in the neural network, weights for the neural network that come with the model.

For the different deep learning models we used different sets of training and testing images. For the Inception and ResNet models, the training set contained 211 images and testing set 36 images (Table 4). For the MobileNet model, the first run using the initial split into training and testing sets (Table 4) did not yield a good accuracy. Therefore, we increased the size of the training set to improve the accuracy, because the model needed more images for training: 237 images used in the training set and 10 images in the testing set (at least 1 for each snake species). For the VGG16 model, we trained on 230 images and tested 50 images. This model gave a prediction answer based on the Boolean true or false parameters, meaning whether an image is classified correctly or incorrectly. We used a few of the training images for testing to verify if the model was correctly predicting the trained images to get false positives or false negatives.

#### 2.3.4. Modeling

**Figure 7:**
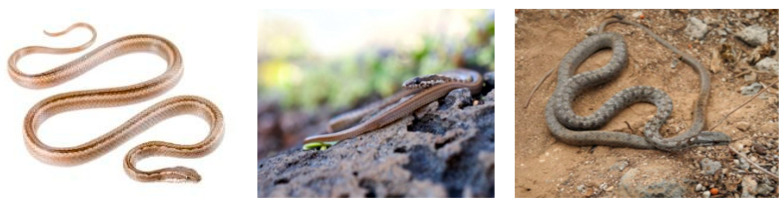
Three different variations of *P. biserialis* [5,31,32]. Images demonstrate a variety in background and percentage of portion covered by a snake in the entire frame.

All images varied in size and features. In some instances, the snakes were the object in focus and the background was blurry. Thus, the area occupied by the object was different in each image. For instance, the images of *Pseudalsophis biserialis* show the object in three different variations (Figure 7).

Therefore, the question is how to process the image in neural networks and obtain the result with high accuracy? One solution is to make the network deeper by adding a greater number of layers in the network, but sometimes very deep networks are prone to overfitting. Choosing the right kernel size for the neural network becomes a challenging task. Usually “a larger kernel is preferred for information that is distributed more globally, and a smaller kernel is preferred for information that is distributed more locally” [20].

## 3. Results

Google has released few object-detection APIs (application programming interface) for TensorFlow, an open source platform for machine learning that has prebuilt architectures and weights for specific models of neural networks. From TensorFlow, we used Faster R-CNN Inception and R-CNN ResNet models for the object detection and image classification in the snake images.

VGG16 network is loaded in the Keras deep learning library in TensorFlow. Keras has the advantage of containing pre-trained models (such as VGG16) which we used and modified for our classification task. Oxford Visual Geometry Group provides weights, but due to their size, the model requires a lot of time and memory to run.

While using four different modified R-CNN architectures, Inception V2, ResNet, MobileNet, and VGG16, to implement object detection and image recognition of nine snake species of *Pseudalsophis*, the models demonstrated the following performance results. MobileNet gave the lowest accuracy of 10%, Inception V2 model demonstrated performance around 70%, whereas, both VGG16 and ResNet tied for 75% accuracy (Table 5). Pretrained models were used to test the benefits of each individual algorithm, however, for future research, to achieve better accuracy, the models should be trained with a much bigger data set of images. One of the complications of our work was that we did not have enough images for some species to train these species correctly. To further improve the accuracy, we will need to obtain more images of each species to train the model. In the subsections below, we discuss the performance of each model in more detail.

### 3.1. Inception V2

We used Faster R-CNN Inception V2 to train the model. The model was trained on all nine species of snakes. Loss started at around loss = 3 at step 0, and we continued to train our model until we obtained a loss around 0.08 and below. It took 7000 steps to obtain that loss and 14 to 15 h to train the model. Once this loss stayed below 0.08 constantly, we stopped training the model and generated the inference graph for the model that we used in our code to test the images. The inference graph was generated from the highest checkpoint that was saved while running the model.

**Figure 8:**
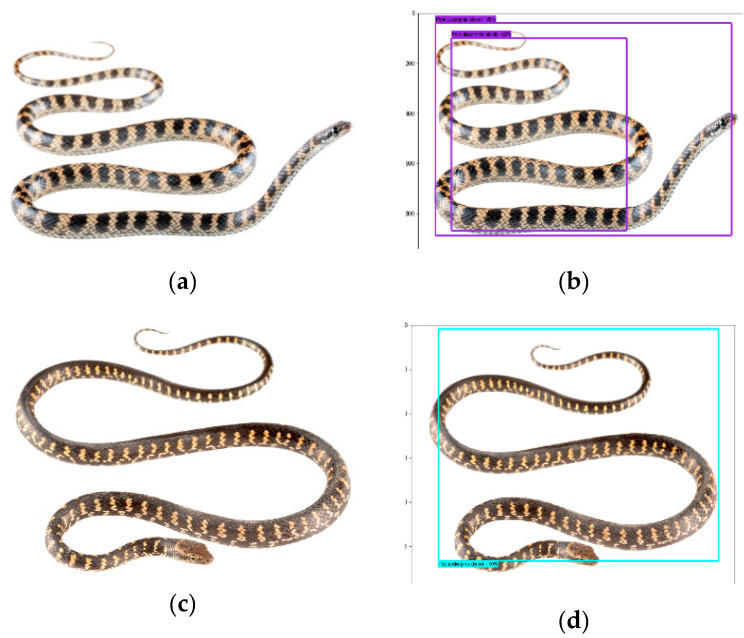
*Pseudalsophis* snake species recognition results obtained while using the Inception V2 model; *P. slevini* correctly classified with 98% probability (**a**,**b**), *P. darwini* correctly classified with 95% probability (**c**,**d**). (**a**,**c**): actual images. (**b**,**d**): prediction output.

While using the Inception V2 model, we identified 70% of the test images correctly with an 80–90% confidence interval for each snake species (Figure 8).

### 3.2. ResNet

We used the Faster R-CNN ResNet V1 model (faster_rcnn_resent_v1_feature_extractor) to train the model. All nine snake species were used to train the model. The step loss started at around loss = 4 at step 0, and we continued to train the model until we obtained the step loss less than 0.05. It took 3500 steps to obtain that loss and 17 h to train the model.

**Figure 9:**
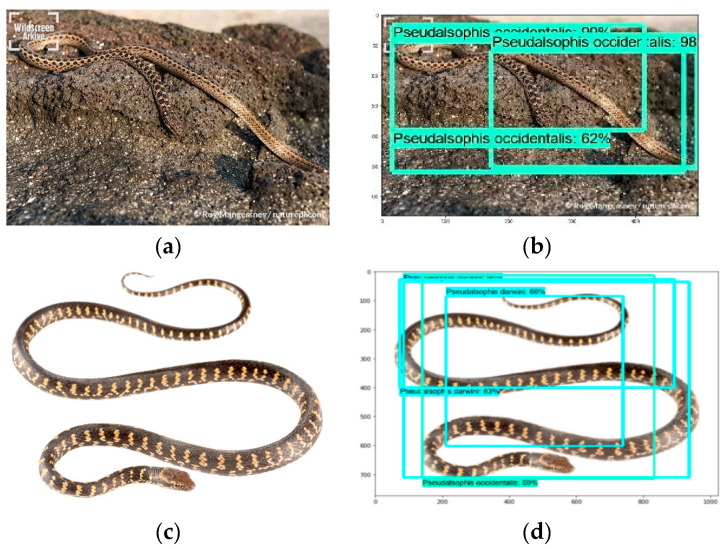
*Pseudalsophis* snake species recognition results obtained while using the ResNet model: 75% of images were correctly recognized. *P. occidentalis* [33] correctly classified with 98% probability (**a**), (**b**). *P. darwini* in the same image (**c**), (**d**) was classified correctly with 83% probability, and incorrectly classified as *P. occidentalis* with 59% probability. (**a**,**c**): actual images. (**b**,**d**): prediction output.

While using the ResNet, 75% of the images were identified correctly with a 95–99% confidence interval for each snake species. Individuals of *P. occidentalis* in the images below were identified correctly with a 98% confidence interval (Figure 9).

Besides correctly identifying most of the images, it is worth mentioning the failure example of our implemented ResNet model. The ResNet model identified incorrectly individuals of *P. darwini* as *P. occidentalis* (Figure 9) by predicting both species in one image.

### 3.3. MobileNet

The MobileNet model was trained on all nine species of snakes. The model training time was very long, taking almost 20 h to bring the step loss to less than 0.5, which started at 14.0 for MobileNet. Unfortunately, with 10 images used for testing, all the images were classified as *P. thomasi*, giving us an accuracy of 10%, considering only *P. thomasi* was correctly classified. The neural network failed for this particular model. However, given more time and resources this model can learn deeply and can be improved.

### 3.4. VGG16

The VGG16 model was trained and tested on all nine species of snakes. The process was very fast, taking only 20 min to run the code because the images were trained on the last layer only. With 50 images used for testing, 13 out of the 50 images were predicted incorrectly. *P. occidentalis* and *P. biserialis* were the two species that were misclassified in the 13 images, the model predicted both species in one image. Therefore, this model gave us an accuracy of 75%. Some complications that appeared while training and testing this model were that it had difficulties in recognizing certain species of snakes in images with a cluttered background. Accordingly, it caused difficulties when distinguishing the differences in snake species.

## 4. Discussion

This research project succeeded in implementing different models in recognizing snake species based on image data, though there is room for improvement. The following are some of the challenges faced by this project:

1. Image dataset was limited. There were not enough images available for each species to train the model. Two hundred thirty images to train a deep learning model is not enough to achieve good accuracy. Image data augmentation could be a remedy in this case, though it requires additional deep-domain skills in deep learning, image processing, and computer vision. Image data augmentation would allow us to significantly increase the data set size and diversity of images, providing the options of image modification by turning, flipping, padding, cropping, brightening, or darkening the object and background in the image.
2. There were a few images with complex backgrounds in which separating the snake from its surroundings was difficult. To resolve this issue, removing the backgrounds in images would significantly improve the results, however, the solution to automate the background removal for hundreds of thousands of images would have to be provided. Recent scientists’ achievements reveal that there is a promising background removal method called DOG, created by Fang et al. [34,35], used alongside VGG-DCGAN to increase the effectiveness of image classification. The method is based on CNN and created from the original model Tiramisu (One Hundred Layers Tiramisu) [36], however, with fewer layers leading to a better performance. Lastly, transfer learning can be utilized to deal with complex backgrounds in images, which allows for incorporation of knowledge previously gained from other models into current models. This would improve the accuracy and performance of the model, since it is leveraging knowledge gained from previous trained models.

### 4.1. Future Scope: From a Data Science Perspective

There are further pretrained models and CNN architectures trained on datasets, such as CIFAR 10 or CIFAR 100, that can be used to train the Galápagos snake species images. YOLO is one of the promising models. YOLO (you only look once) algorithm is different from other object detection models because it uses a single convolutional neural network for both classification and localizing the object using bounding boxes [37]. Previous algorithms used region-based techniques, while YOLO uses the whole image for detection [37]. YOLO divides the imagine into an S × S grid. If an object of interest falls into one of the grids, that grid is responsible for detecting that object. Each grid has a confidence score for predicting its object, but if there is no object shown in that grid, the confidence score is zero. YOLO is based on the Darknet, which is an open source neural network framework in C language. However, it was translated from Darknet to Darkflow to allow for its use in TensorFlow in Python for deep learning. The main benefit of this algorithm is that its processing and speed detection is rapid, which is excellent for real-time processing [37].

The research project presented in this paper was developed on pre-built structures of the TensorFlow and Keras models, such as R-CNN ResNet, R-CNN Inception, and MobileNet. Our future step will be to build our own architecture and convolutional layers to achieve better accuracy. There are many deep learning studios and APIs, such as Deep Learning Studio launched by Deep Cognition, that can help build each layer in the neural network as we aim to improve the outcome.

Another library or package built by Facebook-PyTorch provides a high level of tensor computation with strong GPU acceleration and a deep neural network built on a tape-based autograd system. It can be used to identify the snake species in our dataset.

### 4.2. Future Scope: From a Software Development Perspective

**Figure 10:**
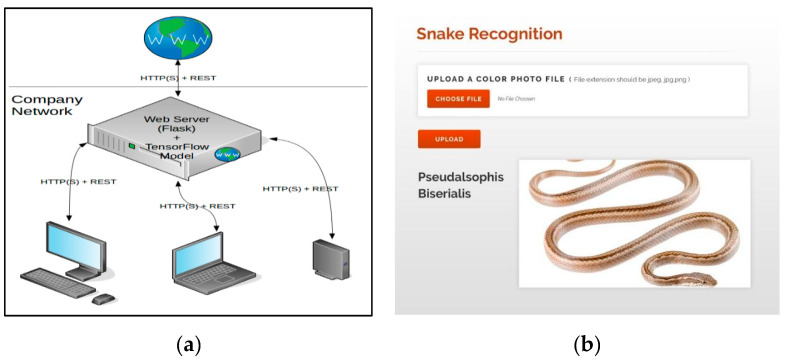
Artificial intelligence software application demo for automated real-time decision making software, helping visitors and park rangers in the Galápagos islands to correctly identify the snake species that they see in nature. (**a**): architecture and network connectivity of the artificial intelligence software application. (**b**): predicted output “Pseudalsophis Biserialis” after a user uploads a snake photo into the artificial intelligence software application.

The final target of this research project was to build an artificial intelligence (AI) platform, in terms of a web or mobile application, that would serve as a real-time decision making mechanism for the visitors and park rangers in the Galápagos islands. A user would take a picture and upload it to the AI platform to check for the correct snake species name. Our developed deep learning model, that was built using TensorFlow, is connected via an API with the Python backend and frontend technologies. Flask is the Python library that allowed us to build the web application by connecting TensorFlow to the machine learning algorithm. The demo version of the AI application software is presented in Figure 10. Image testing took less than 15 s to identify an image. Even though, the testing time was short for an image detection/recognition task, we believe it can be reduced by using new technologies, described here, to improve the real-time product–user interaction. The final step of the AI application software not only gives the Galápagos national park rangers and tourists the class of the predicted snake species, but also educates users by providing them with information about the distribution, natural history, conservation, and etymology of the snake.

## Figures and Tables

**Table 1:** Different color spaces with color descriptions and their classification basis.

Color Space	Color Description	Classification Based On
RGB	Moments of R channel	Lightness
XYZ	Moments of Z channel	Lightness, blue color
xyz	Moments of x channel	Red color
xyz	Moments of z channel	Blue color
YCbCr	Moments of Y channel	Lightness
YCbCr	Moments of Cb channel	Blue color
YCbCr	Moments of Cr channel	Red color
HSV	Moments of H channel	Number of colors
HSV	Moments of S channel	Sharp and blurred colors
HSV	Moments of V channel	Lightness
Opponent space	Moments of 1 channel	Blue and red colors
Opponent space	Moments of 2 channel	Blue color, sharp and blurred colors
RGB	Moments	Lightness, blue color
YCbCr	Moments	Lightness, blue color
HSV	Moments	Darkness, blue color
rgb	Histogram	Blue and green colors
rgb	CCV	Lightness
xyz	CCV	Lightness, blue and green colors
YCbCr	CCV	Blue color
Opponent space	CCV	Blue color

**Table 2:** Performance of algorithms used for snake species classification in Perlis park, Malaysia [11].

Classifier	Correct Prediction	Recall	Precision	F-Measure
Naïve Bayes	75.64	0.92	0.94	0.93
Backpropagation neural network	87.93	1	0.99	0.99
Nearest neighbors	89.22	1	0.96	0.97
K-NN (k = 7)	80.34	1	0.96	0.97
Decision tree J48	71.29	0.79	0.71	0.72

**Table 3:** External parameters (input) for training the models.

File Name	Width	Height	Class	x_min_	y_min_	x_max_	y_max_
14924394811_c505bc3d_o.jpg	3497	2332	*P. biserialis*	1002	625	3497	2015
151761_5-Galapagos.jpg	600	500	*P. occidentalis*	41	65	554	442
16081314279_c833e990_b.jpg	1024	683	*P. slevini*	164	269	698	412
182611421_cba87acd82_o.jpg	3648	2736	*P. biserialis*	449	673	3166	2166

**Table 4:** Number of images used for training and testing sets for Inception and ResNet models.

*Pseudalsophis* Snake Species	Training Set Images	Test Set Images	Total Images
*P. biserialis*	27	5	32
*P. darwini*	10	2	12
*P. dorsalis*	23	4	27
*P. hephaestus*	8	1	9
*P. hoodensis*	23	3	26
*P. occidentalis*	58	10	68
*P. slevini*	6	1	7
*P. steindachneri*	16	3	19
*P. thomasi*	40	7	47
Total:	211	36	247

**Table 5:** Summary of performance results for all region-based convolutional neural network (R-CNN) models implemented in this research.

R-CNN Model	Accuracy
ResNet	Around 75%
Inception V2	Around 70%
VGG16	Around 70%
MobileNet	Around 10%
